# Relationship between salmon egg subsidy and the distribution of an avian predator

**DOI:** 10.1002/ece3.9696

**Published:** 2022-12-28

**Authors:** Taihei Yamada, Hirotaka Katahira, Kazuki Miura, Futoshi Nakamura

**Affiliations:** ^1^ Graduate School of Agriculture Hokkaido University Sapporo Japan; ^2^ Department of Environmental Science, School of Life and Environmental Science Azabu University Sagamihara Japan; ^3^ Shiretoko Museum Shari Japan; ^4^ Present address: Research Institute of Energy, Environment and Geology Hokkaido Research Organization Sapporo Japan

**Keywords:** behavioral ecology, marine‐derived nutrients, Pacific salmon, resource subsidy, Salmonidae, stream ecology

## Abstract

As a spatial subsidy, which is the phenomenon of transferring resources from a donor system to a recipient system, anadromous salmonids contribute to the supply of marine‐derived nutrients to freshwater and terrestrial systems. Live salmon and salmon carcasses and eggs are utilized by various organisms and affect their abundance and distribution. However, the evaluation of the effect of salmon subsidies on the abundance and distribution of terrestrial animals is biased toward predators or scavengers that utilize spawning adults and carcasses, and few studies have focused on the effect of salmon eggs as a subsidy. To avoid underestimating the function of salmon subsidies, the response to the availability of salmon eggs in various systems should be investigated. Here, we investigated the abundance and feeding behavior of the brown dipper *Cinclus pallasii*, as a consumer of salmon eggs, based on the hypothesis that the availability of salmon eggs affects the diet composition and stream distribution of this small predator. In addition, to test whether changes in the abundance of brown dippers are determined by salmon spawning, their abundance was compared upstream and downstream of the check dams in three streams during the peak spawning period. Brown dippers used salmon eggs during the spawning season (53.7% of diet composition), and their abundance increased as the number of spawning redds increased. In contrast, this pattern was not observed upstream of the check dam. These results suggested that the abundance and stream distribution of brown dippers vary according to the variation in the spatiotemporal availability of salmon eggs.

## Graphical abstract

We evaluated the effect of salmon egg subsidy on the distribution and diet composition of brown dippers, which are riparian birds mainly feeding on aquatic invertebrates. Our results showed that salmon eggs are the dominant dietary item for brown dippers during the salmon spawning season and that the abundance and stream distribution of brown dippers can be explained by the availability of salmon eggs.

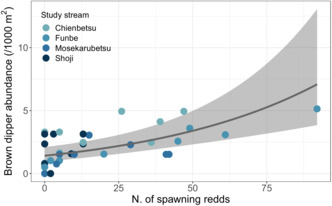

## 1 INTRODUCTION

Spatial subsidies are a phenomenon in which resources are transferred from a donor system to a recipient system (Polis et al., 1997). Spatial subsidies play a crucial role in biological communities because they affect the abundance and distribution patterns of organisms and the food web structure in recipient systems by affecting the availability of basal resources (Hocking et al., 2013; Kawaguchi et al., 2003; Nakano & Murakami, 2001; Spiller et al., 2010; Terui et al., 2018).

Anadromous salmonids, well‐known spatial subsidy representatives, transport marine‐derived nutrients and energy to freshwater and terrestrial ecosystems through their migrations (Gende et al., 2002; Hocking & Reynolds, 2011; Koshino et al., 2013; Schindler et al., 2003). Salmon subsidies contribute to increasing aquatic invertebrate biomass and freshwater fish abundance in rivers (Denton et al., 2009; Wipfli et al., 1998, 1999). Spawning adults and carcasses are also used as food by terrestrial animals not only in the underwater ecosystem but also in surrounding riparian ecosystems and eventually affect the abundance and distribution of terrestrial scavengers and top predators (Boulanger et al., 2004; Christie & Reimchen, 2005; Field & Reynolds, 2013; Levi et al., 2012; Walters et al., 2021). Salmon subsidies thus provide insights into how multiple ecosystems are tangled together.

Past investigations on the effects of salmon subsidies on terrestrial organism abundance and distribution have thus far been biased toward top predators or scavengers that utilize spawning adults and carcasses (e.g., Boulanger et al., 2004; Christie & Reimchen, 2005; Field & Reynolds, 2013; Levi et al., 2012; Walters et al., 2021). Considering that terrestrial organisms are provided multiple resources by salmon runs, such as spawned eggs and fry, as well as spawning adults and carcasses (Munro, 1941; Willson & Halupka, 1995), salmon subsidies may have even more unexpected far‐reaching effects. Spawned eggs can be valuable food for consumers because female salmon allocate much of their lipids into egg development (Hendry & Berg, 1999). Stream salmonids are indeed known to mainly consume salmon eggs during the salmon spawning season (Armstrong & Bond, 2013; Moore et al., 2008; Scheuerell et al., 2007), and their abundance increases with their availability (Denton et al., 2009). The same should be true for terrestrial organisms, where spawned eggs may affect the abundance and distribution of terrestrial organisms that do not utilize spawning adults and carcasses, so responses to salmon egg availability in various systems must be clarified to avoid underestimating the function of salmon subsidies.

**FIGURE 1:**
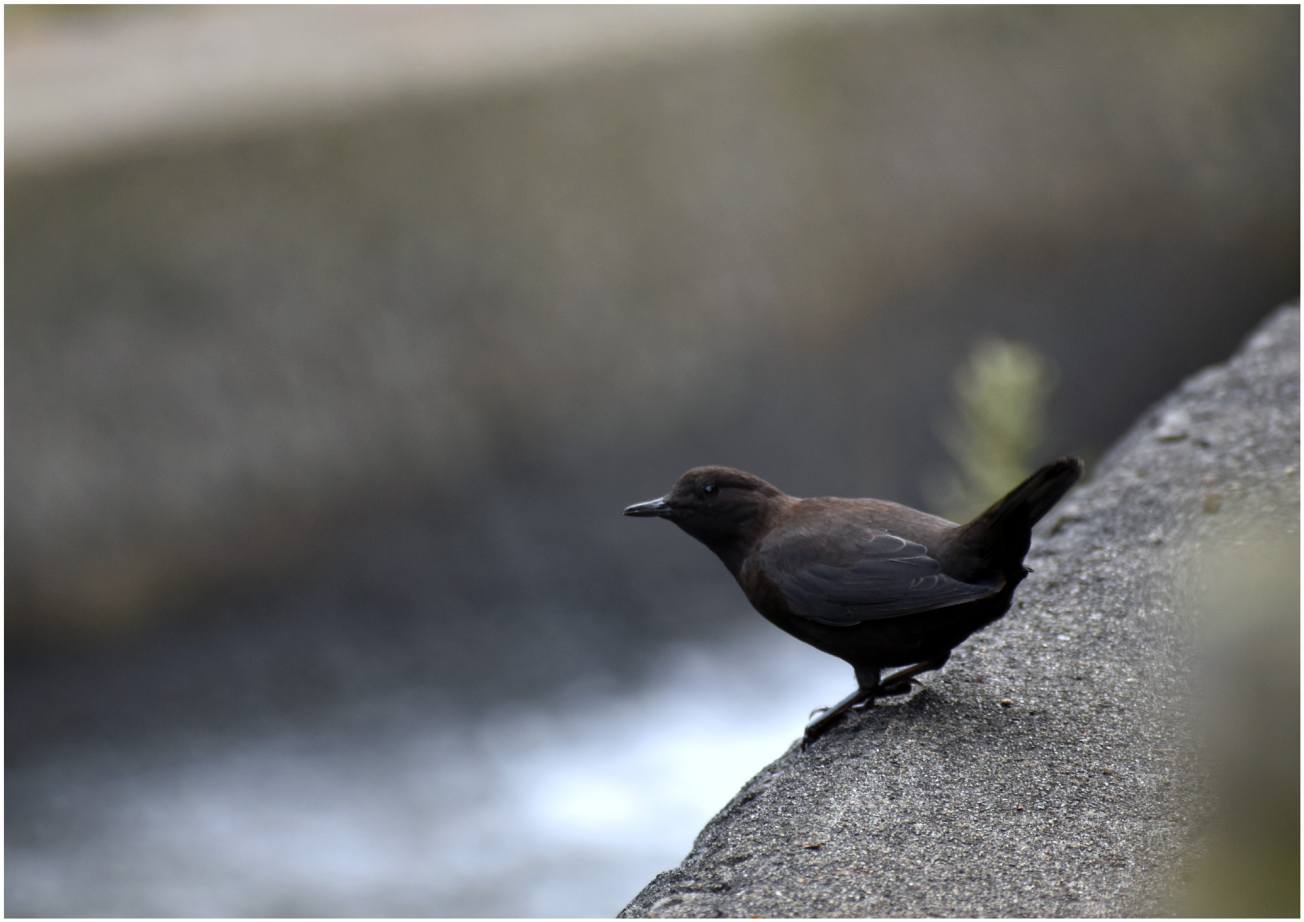
The brown dipper *Cinclus pallasii*. Photo by Yuya Eguchi.

Dippers (Aves: Cinclidae) are riparian birds that mainly feed on aquatic invertebrates by diving into the water (Eguchi, 1990; Taylor & O'Halloran, 1997, 2001) and are known to use salmon eggs in available rivers and seasons (Goodge, 1959; Obermeyer et al., 1999, 2006; Reimchen, 2017; Whitehorne, 2010). For example, the American dipper *Cinclus mexicanus* can achieve higher reproductive success (as measured by fecundity and juvenile growth) in reaches where *Oncorhynchus* spawn than in those where it does not (Obermeyer et al., 2006; Tonra et al., 2016). The population size of the white‐throated dipper *C. cinclus* in Norway may also benefit from eating salmon fry because it was correlated with the annual density of salmon fry (Nilsson et al., 2018). Because dippers, which are not scavengers, are not affected by the amount of carcasses—in addition to their well‐studied relationship with salmon, as noted above—they are a suitable model species for examining the effect of salmon egg subsidies on the abundance and distribution of terrestrial animals. The brown dipper *C. pallasii* (Figure 1), which is distributed in Asia (Hong et al., 2019), preys on salmon eggs and juvenile salmon (Murata, 1900). However, its actual status has never been evaluated quantitatively, and its relationship with salmon subsidies has long been overlooked.

We therefore investigated the abundance and diet composition of the brown dipper in the Shiretoko Peninsula of Hokkaido, northern Japan, where the spawning migrations of salmon are well observed based on the hypothesis that the availability of salmon subsidies drives the diet composition and stream distribution of this small predator. In addition, by comparing the abundance of brown dippers above and below check dams for sediment control where salmon cannot run upstream during peak salmon spawning runs, we tested whether changes in the abundance of brown dippers are determined by salmon spawning. More specifically, it was predicted that salmon spawning would cause a shift in the diet of brown dippers to salmon eggs and an increase in the abundance of brown dippers by altering the distribution of food resources, while no such pattern occurs upstream of the check dam.

## 2 MATERIALS AND METHODS

### 2.1 Study site

**FIGURE 2:**
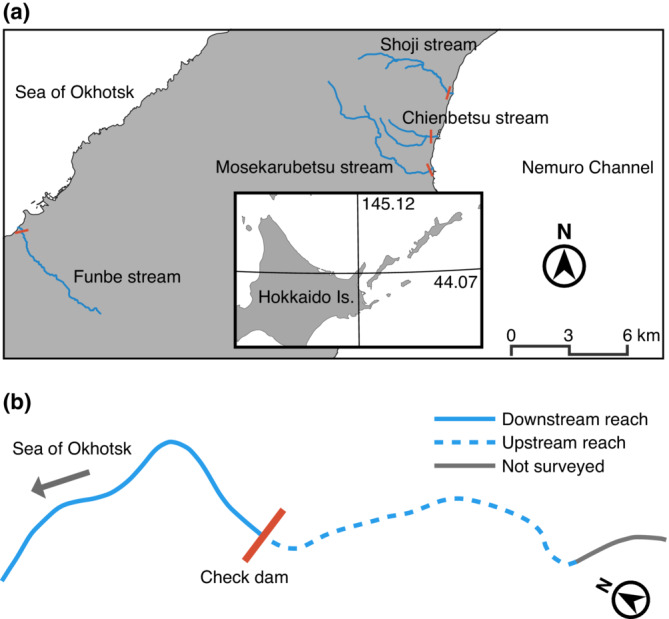
(a) Map of the study area located on the Shiretoko Peninsula. The blue lines indicate the streams surveyed. Orange lines indicate the check dams for sediment control, which are the upper end of the downstream study reaches. (b) Enlarged view of the study section in the Funbe stream as an example to facilitate understanding of the location of the study section.

Four streams located in the Shiretoko Peninsula were selected for the present survey (Figure 2a; Table 1). Natural spawning sustains pink salmon *Oncorhynchus gorbuscha* and chum salmon *O. keta* populations in these streams, and the former has a higher number of runs (T. Yamada, unpublished data). The release of juvenile chum salmon has been conducted only in the Mosekarubetsu stream, and in‐stream harvesting does not occur in all streams. The central part of the Shiretoko Peninsula has been designated as a World Natural Heritage site since 2005, partially because of the close relationships between the marine and terrestrial ecosystems sustained by the anadromous migration of pink salmon and chum salmon (IUCN, 2005). The upper reaches of the studied streams are included in the Shiretoko World Natural Heritage site. Rivers and streams in the Shiretoko Peninsula are highly fragmented by more than 330 artificial dams (Takahashi et al., 2005), which is no exception in all selected streams. Study sections were set up in each stream from the mouth of the stream to the proximal check dam for sediment control (Figure 2a; Table 1). The Chienbetsu stream was surveyed up to the second proximal check dam because many salmon pass through the first proximal check dam. In addition, to examine the brown dipper abundance in the reaches without salmon subsidies at the peak spawning period, we established an additional study section up to 400 m from the check dam, which is the upper end of the downstream study section in each stream, except in the Chienbetsu stream, which rarely runs upstream but has a fishway at the second proximal check dam allowing migration to the upper reaches (Figure 2a,b; Table 1). The distance between the upper end of the study section downstream of the check dam and the lower end of the study section upstream of the check dam in each stream was 1–2 m (Figure 2b). We also measured the stream surface area of all study sections only once in each section during the study period.

**TABLE 1:** Physical characteristics for each study section

Study section	Length (m)	Width (m)	Elevation (m)	Slope (%)
Chienbetsu stream				
Downstream	289.4	4.1	12.1	18.3
Funbe stream				
Upstream	400.0	6.8	35.6	14.6
Downstream	348.2	5.9	12.3	19.4
Mosekarubetsu stream				
Upstream	400.0	6.5	23.8	14.1
Downstream	154.9	8.7	7.4	10.4
Shoji stream				
Upstream	400.0	7.1	28.6	31.9
Downstream	211.3	6.2	10.9	32.8

*Note*: Length (m) is the total length of the study reach. Width (m) is the average of the widths measured on 10 transects. Elevation (m) and slope (%) are the averages calculated based on a 10 m digital elevation model provided by the Geospatial Information Authority of Japan.

### 2.2 Field survey

**FIGURE 3:**
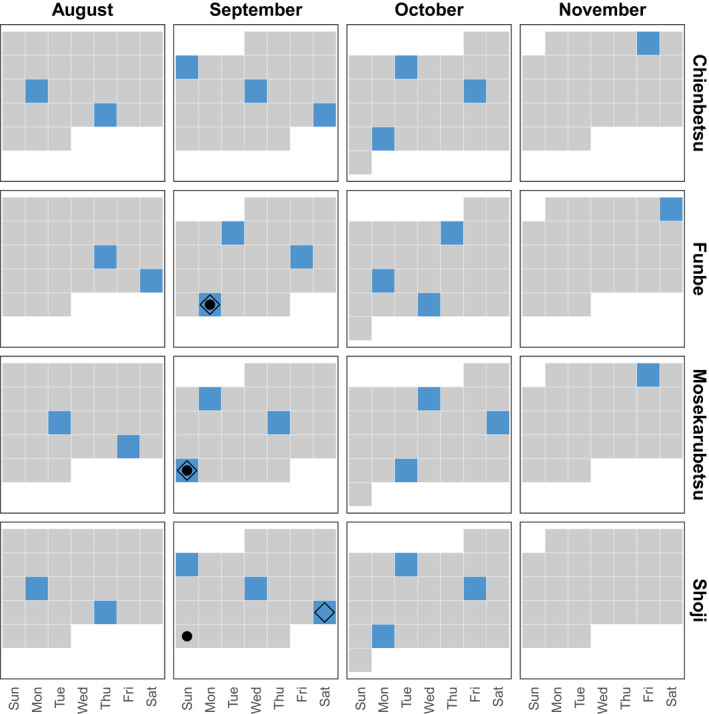
Sampling schedule for each stream. Squares filled with blue indicate the date on which the routine abundance and diet surveys were conducted. Solid circles and open rhombuses denote the dates when abundance data were obtained upstream and downstream of the check dam, respectively.

Temporal changes in the abundance and diet of brown dippers were evaluated from mid‐August to early November 2021, the spawning period of pink salmon. Field observations were conducted in one or two streams per day for a total effort of 8 or 9 days at 9‐ to 11‐day intervals in each stream (Figure 3). In the observation protocol, one investigator (T. Yamada) walked along the study section from the lower to the upper reach, counting the number of brown dippers. To avoid recounting birds, the investigator checked where the flying individuals stopped and ignored individuals who flew ahead of the investigator, expecting territorial individuals to characteristically “double‐back” when pushed to the ends of their territory (Chiu et al., 2008).

On the same day, after the count survey, an investigator re‐walked the study section to conduct the diet survey. In the diet survey, when the investigator found an individual, they approached it at an observable distance and recorded the diet composition and age category (adult or juvenile) using binoculars (MONARCH 10 × 42; Nikon) (Obermeyer et al., 1999). The age category was classified by the presence or absence of juvenile plumage. The contents of the dipper's diet were classified into four categories: aquatic insects, terrestrial insects, algae, and salmon eggs. We recorded the number of predated individual eggs and invertebrates, but algae were recorded as the number of times feeding. If no observable individuals were found, no observations were made. The diet survey was conducted only once per individual at each observation cycle; the mean ± SD observation time was 4.29 ± 3.16 min.

Salmon spawning redds were also visually counted on the same day as the above observation procedures to obtain a potential index of the availability of salmon eggs. Pink salmon exhibit “probing,” a periodic and short‐term migration behavior between the sea and multiple drainages (Morita, 2021; Thedinga et al., 2000). If many individuals exhibit probing, salmon abundance cannot be a direct indicator of the number of spawners; therefore, we used the number of spawning redds as an indicator of the number of spawners. Spawning redds were visually judged as the area of disturbed gravel or bright (denuded) areas among the periphyton‐covered gravel (Ortlepp & Mürle, 2003; Pedersen et al., 2009). In addition to counting spawning redds, an investigator walked in the upstream direction and visually counted all live salmon. All counting was conducted when the water visibility was good enough to see the bottom of the riffles.

The abundance of brown dippers was also surveyed upstream of check dams in three streams other than the Chienbetsu stream at the end of September 2021 during the peak spawning period of pink salmon (Figure 3). An investigator walked from the check dam to the upstream end of the study section counting the number of dippers, as was the case in the lower reach survey. This survey was conducted in the fifth cycle of the count survey mentioned above (Figure 3).

### 2.3 Statistical analysis

The dependence on salmon eggs in brown dipper diets was evaluated by fitting a generalized linear mixed model (GLMM) to the individual diet data. In the analysis, the ratio of salmon eggs in the diet composition (number of salmon eggs in the diet/total number of feeds; hereinafter called “salmon egg ratio”) was used as a response variable, and it was assumed to follow a binomial distribution with a logit link function. Age category and number of spawning redds were used as the candidate explanatory variables, considering stream ID and observation cycle ID as nested random intercepts. To avoid multicollinearity, variance inflation factors (VIFs) were calculated before the analysis; all variables had values <2.5, the threshold indicative of troubling collinearity for regressions (Johnston et al., 2018). The significance of the explanatory variables was evaluated using type II Wald chi‐square tests (*p* < .05). We applied a backward selection method using *p* values by dropping out non‐significant effects. We also tested for temporal autocorrelation and overdispersion by the testTemporalAutocorrelation and testDispersion functions from the DHARMa package (Hartig, 2022), respectively. The final model had no evidence of temporal autocorrelation or overdispersion.

A relationship between the availability of salmon eggs (represented as the number of spawning redds) and the brown dipper abundance was also estimated by fitting GLMMs to the dipper count data as a response variable assuming a Poisson distribution with a log link function. The number of spawning redds and time on the day when the survey was started were used as the candidate explanatory variables, considering log‐transformed stream surface area as an offset term and stream ID as a random intercept. The VIFs of all variables were <2.5. The significance of variables was determined using the same methods described above. We also detected no temporal autocorrelation or overdispersion in the final model.

Since resource availability may affect organism distribution (e.g., Dingle, 2014; Dingle & Drake, 2007), it was also expected that the dipper abundance differed between the upper and lower reaches of the check dam. Brown dipper abundance in the fifth observation cycle at three streams (Funbe, Mosekarubetsu, and Shoji streams) was compared between the lower reaches and the upper reaches of the check dams by fitting the count data to a GLMM assuming a Poisson distribution with a log link function considering log‐transformed stream surface area as an offset term and the stream ID as a random intercept. The same method as above was used to evaluate the significance of the variable. No overdispersion was detected in the final model.

All data analyses were conducted with R v. 4.2.0 (R Core Team, 2022) using lme4 v. 1.1.30 (Bates et al., 2015) for GLMMs, car v. 3.0.13 (Fox & Weisberg, 2019), for Wald chi‐square tests, and DHARMa v. 0.4.6 (Hartig, 2022) for testing temporal autocorrelation and overdispersion.

## 3 RESULTS

A total of 108 brown dipper individuals, 631 redds, 1257 pink salmon individuals, and 118 chum salmon individuals were observed during our survey. Feeding behavior was monitored in four individuals (three adults and one juvenile) in the pre‐spawning period and in 24 individuals (15 adults and nine juveniles) in the spawning period from the three streams, except for the Mosekarubetsu stream, where close observation could not be made.

**FIGURE 4:**
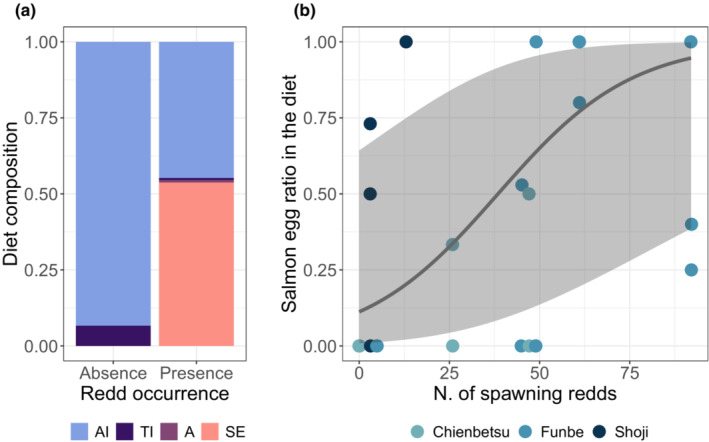
(a) Composition of brown dipper diets during the salmon pre‐spawning and spawning periods. A, algae; AI, aquatic invertebrate; SE, salmon egg; TI, terrestrial invertebrate. (b) Mean predicted marginal effects of the number of spawning redds on the salmon egg ratio in brown dipper diets. The shaded area indicates the 95% CI.

The diet composition changed between the salmon pre‐spawning and spawning periods (Figure 4a). The percentage of salmon eggs in the diet reached 53.7% (Figure 4a) during the latter period. The brown dippers ingested only small food in the water and did not peck salmon carcasses. As a result of the type II Wald chi‐square test, only the number of spawning redds was significant (*χ* ^2^ = 7.28, *p* = .007) in the salmon egg ratio model, indicating that the number of spawning redds had a positive effect on the salmon egg ratio in the diet (Marginal *R* ^2^ = .319, Conditional *R* ^2^ = .494; Figure 4b; Table 2).

**TABLE 2:** Results of GLMMs testing the effects of the number of redds on the salmon egg ratio in brown dipper diets and brown dipper abundance and the presence/absence of salmon (below/above the check dam) on brown dipper abundance during the peak spawning period

Fixed effect	Estimate	SE	*z* value	*p*
Salmon egg ratio in dipper diets				
Intercept	−2.07	1.36	−1.53	.1270
Number of redds	0.05	0.02	2.70	**.0070**
Seasonal dipper abundance				
Intercept	−6.56	0.20	−32.47	**<.0001**
Number of redds	0.02	0.00	4.40	**<.0001**
Comparison of dipper abundance upstream and downstream of dam				
Intercept	−8.32	0.72	−11.52	**<.0001**
Section—below dam	2.63	0.75	3.50	**.0005**

*Note*: Bold indicates that the variable is significant (*p* < .05).

**FIGURE 5:**
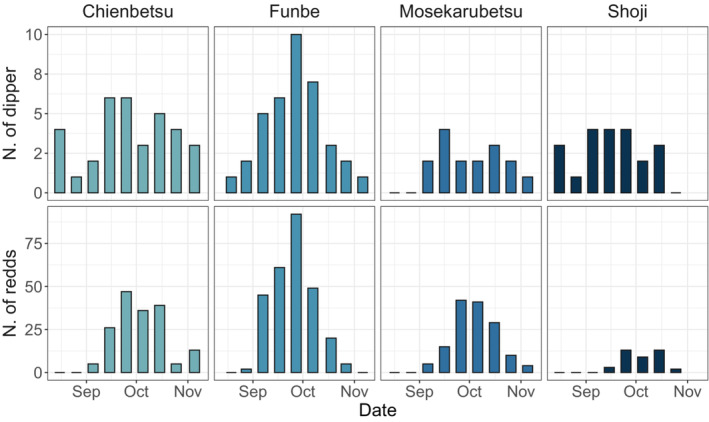
Observed brown dipper abundance and number of spawning redds in relation to survey date in each stream.

**FIGURE 6:**
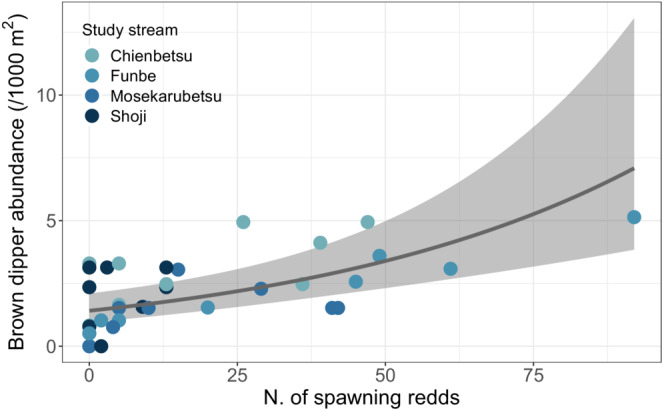
Mean predicted marginal effects of the number of spawning redds on brown dipper abundance. The 95% CI is denoted by the shaded area.

**FIGURE 7:**
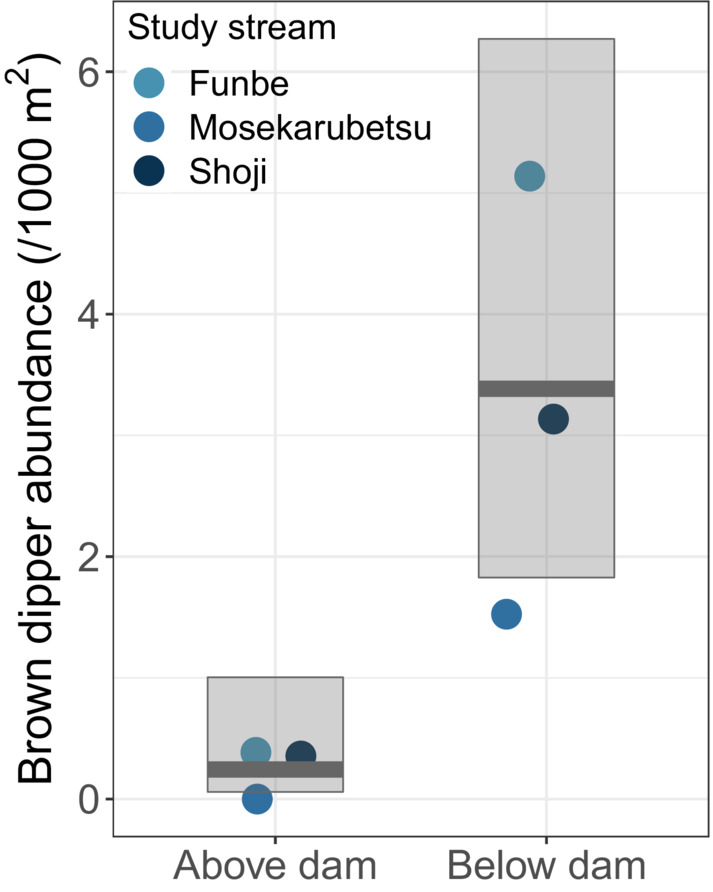
Mean predicted marginal effects of salmon occurrence on brown dipper abundance during the peak spawning season. The translucent boxes indicate 95% CIs.

The abundance survey showed that brown dipper abundance tended to fluctuate in response to the number of salmon redds (Figure 5). In the Wald chi‐square test, only the number of spawning redds was significant (*χ* ^2^ = 19.32, *p* = .00001). The brown dipper abundance was positively correlated with the number of spawning redds in the selected model (Marginal *R* ^2^ = .396, Conditional *R* ^2^ = .528; Figure 6; Table 2). The comparison between abundances in the upper and lower sections of the check dam was significant (*χ* ^2^ = 12.27, *p* = .0005) and showed that the abundance in the lower section was significantly higher than that in the upper section (Marginal *R* ^2^ = .792, Conditional *R* ^2^ = .821; Figure 7; Table 2).

## 4 DISCUSSION

This study demonstrates for the first time that salmon eggs are the dominant dietary item for the brown dipper during the salmon spawning season and that the abundance and stream distribution of terrestrial vertebrate species can be predicted by the number of spawning redds used to represent the availability of salmon eggs. In addition, brown dipper abundance during the peak spawning season differed significantly between the upstream and downstream of the check dam, with the downstream abundance being higher. This disproportional dipper abundance between the upstream and downstream of check dams can be attributed to the spatial variation in salmon egg availability during the spawning season caused by the check dams. It is therefore indicated that the distribution of brown dippers varies according to variations in the spatiotemporal availability of salmon subsidies. Further studies examining the dipper distribution upstream and downstream of check dams before the spawning season are critical to reinforcing our discussion.

The effects of salmon subsidies can be divided into two pathways: direct effects as food for consumers and indirect effects through various bottom‐up interactions. Several studies have indicated that salmon subsidies indirectly affect the territory size, habitat selection, abundance, and diversity of songbirds (Christine & Reimchen, 2008; Gende & Willson, 2001; Wagner & Reynolds, 2019; Wilcox et al., 2021), but the direct effect of salmon eggs as subsidies on songbird distribution has been overlooked. Our results provide critical evidence that salmon subsidies have a direct effect on the distribution of songbirds, suggesting that salmon subsidies affect songbirds both indirectly and directly. In the case of freshwater fishes, the direct effect of the salmon egg subsidy has often been examined; stream salmonids consume mainly salmon eggs during the salmon spawning season (Armstrong & Bond, 2013; Moore et al., 2008; Scheuerell et al., 2007), increasing their abundance (Denton et al., 2009). These results are consistent with our results; therefore, it is considered that the salmon egg subsidy is an important resource across taxonomic groups.

While salmon eggs serve as an important food source for brown dippers, the salmon spawning behavior of digging up the riverbed leads to a reduction in the abundance of aquatic invertebrates that are prey for brown dippers (Minakawa & Gara, 2003; Moore & Schindler, 2008). Since the energy value per salmon egg is higher than that per individual aquatic invertebrate (Obermeyer et al., 2006; Whitehorne, 2010), the positive effect of egg eating may outweigh the negative effect of reduced eating of aquatic invertebrates. In fact, juvenile weight and mortality in the American dipper in salmon spawning reaches are known to be higher and lower, respectively, than those in the non‐spawning reaches (Obermeyer et al., 2006). Further verification is required to clarify whether these findings may be supported in the present system.

The abundance of aquatic invertebrates varies greatly with season (Rundio & Lindley, 2008) and declines with flooding (Chiu et al., 2008; McMullen & Lytle, 2012). Accordingly, the decrease in aquatic invertebrate abundance leads to a decline in brown dipper abundance and survival (Chiu et al., 2008, 2013). Pink salmon, chum salmon, and masu salmon running up Japanese rivers spawn during the summer, fall, and winter (Iida et al., 2021; Kovach et al., 2012; Kuzishchin et al., 2009; Quinn, 2018). Since summer and fall are typhoon seasons in East Asia, salmon subsidies may compensate for the decline in aquatic invertebrates. In addition, dippers sometimes prey on salmon fry (Obermeyer et al., 2006; Ormerod, 1985; Ormerod & Tyler, 1986, 1991). Since most salmon fry mainly emerge during spring and summer (Kirillov et al., 2018; Pavlov et al., 2008; Yamada et al., 2022), salmon fry may be used as a food resource by dippers during this period. Therefore, spawning by anadromous salmonids may compensate for declines in the abundance of aquatic invertebrates during various seasons.

Salmon spawning abundance is disturbed by several human activities, such as dam construction and fisheries (Finney et al., 2000; Nakamura & Komiyama, 2010; Romakkaniemi et al., 2003). Although we could not examine the effect of spawner abundance on egg availability in this study, the egg availability for dippers could change with spawner density, as indicated by Moore et al. (2008). In addition, this study shows for the first time that the distribution patterns of small terrestrial predators are determined by the supply of salmon eggs, indicating that the disruption of natural spawning may have unexpected effects on the abundance and distribution of terrestrial salmon egg consumers. However, we have only one set of abundance data for each stream for comparison above and below the check dam and did not consider the differences in environmental conditions between sections; therefore, the effects of fragmentation in this study should be interpreted with caution, and the limitations should be considered. Future studies are needed to closely examine the effects of these anthropogenic restrictions (including stream fragmentation) of salmon egg subsidies on the abundance and distribution of terrestrial consumers.

## AUTHOR CONTRIBUTIONS

**Taihei Yamada:** Conceptualization (equal); data curation (lead); formal analysis (lead); funding acquisition (equal); investigation (lead); visualization (lead); writing – original draft (lead). **Hirotaka Katahira:** Conceptualization (equal); writing – review and editing (equal). **Kazuki Miura:** Conceptualization (equal); writing – review and editing (equal). **Futoshi Nakamura:** Funding acquisition (equal); supervision (lead); writing – review and editing (equal).

## CONFLICT OF INTEREST

None declared.

## Data Availability

All datasets used in this study are available at the Figshare repository: https://doi.org/10.6084/m9.figshare.20341956.v2.

## References

[ece39696-bib-0001] Armstrong, J. B. , & Bond, M. H. (2013). Phenotype flexibility in wild fish: Dolly Varden regulate assimilative capacity to capitalize on annual pulsed subsidies. Journal of Animal Ecology, 82(5), 966–975. 10.1111/1365-2656.12066 23510107

[ece39696-bib-0002] Bates, D. , Mächler, M. , Bolker, B. , & Walker, S. (2015). Fitting linear mixed‐effects models using lme4. Journal of Statistical Software, 67(1), 1–48. 10.18637/jss.v067.i01

[ece39696-bib-0003] Boulanger, J. , Himmer, S. , & Swan, C. (2004). Monitoring of grizzly bear population trends and demography using DNA mark–recapture methods in the Owikeno Lake area of British Columbia. Canadian Journal of Zoology, 82(8), 1267–1277. 10.1139/z04-100

[ece39696-bib-0004] Chiu, M. C. , Kuo, M. H. , Hong, S. Y. , & Sun, Y. H. (2013). Impact of extreme flooding on the annual survival of a riparian predator, the Brown Dipper *Cinclus pallasii* . Ibis, 155(2), 377–383. 10.1111/ibi.12035

[ece39696-bib-0005] Chiu, M. C. , Kuo, M. H. , Sun, Y. H. , Hong, S. Y. , & Kuo, H. C. (2008). Effects of flooding on avian top‐predators and their invertebrate prey in a monsoonal Taiwan stream. Freshwater Biology, 53(7), 1335–1344. 10.1111/j.1365-2427.2008.01968.x

[ece39696-bib-0006] Christie, K. S. , & Reimchen, T. E. (2005). Post‐reproductive Pacific salmon, *Oncorhynchus* spp., as a major nutrient source for large aggregations of gulls, *Larus* spp. The Canadian Field‐Naturalist, 119(2), 202–207. 10.22621/cfn.v119i2.107

[ece39696-bib-0007] Christine, K. S. , & Reimchen, T. E. (2008). Presence of salmon increases passerine density on Pacific Northwest streams. Auk, 125(1), 51–59. 10.1525/auk.2008.125.1.51

[ece39696-bib-0008] Denton, K. P. , Rich, H. B. , & Quinn, T. P. (2009). Diet, movement, and growth of Dolly Varden in response to sockeye salmon subsidies. Transactions of the American Fisheries Society, 138(6), 1207–1219. 10.1577/t09-006.1

[ece39696-bib-0009] Dingle, H. (2014). Migration: The biology of life on the move. Oxford University Press. 10.1093/acprof:oso/9780199640386.001.0001

[ece39696-bib-0010] Dingle, H. , & Drake, V. A. (2007). What is migration? Bioscience, 57(2), 113–121. 10.1641/B570206

[ece39696-bib-0011] Eguchi, K. (1990). The choice of foraging methods of the Brown Dipper, *Cinclus pallasii* (Aves: Cinclidae). Journal of Ethology, 8(2), 121–127. 10.1007/BF02350282

[ece39696-bib-0012] Field, R. D. , & Reynolds, J. D. (2013). Ecological links between salmon, large carnivore predation, and scavenging birds. Journal of Avian Biology, 44(1), 9–16. 10.1111/j.1600-048X.2012.05601.x

[ece39696-bib-0013] Finney, B. P. , Gregory‐Eaves, I. , Sweetman, J. , Douglas, M. S. V. , & Smol, J. P. (2000). Impacts of climatic change and fishing on Pacific salmon abundance over the past 300 years. Science, 290(5492), 795–799. 10.1126/science.290.5492.795 11052941

[ece39696-bib-0014] Fox, J. , & Weisberg, S. (2019). An R companion to applied regression (3rd ed.). Sage.

[ece39696-bib-0015] Gende, S. M. , Edwards, R. T. , Willson, M. F. , & Wipfli, M. S. (2002). Pacific salmon in aquatic and terrestrial ecosystems. Bioscience, 52(10), 917–928. 10.1641/0006-3568(2002)052[0917:PSIAAT]2.0.CO;2

[ece39696-bib-0016] Gende, S. M. , & Willson, M. F. (2001). Passerine densities in riparian forests of southeast Alaska: Potential effects of anadromous spawning salmon. The Condor, 103(3), 624–629. 10.1093/condor/103.3.624

[ece39696-bib-0017] Goodge, W. R. (1959). Locomotion and other behavior of the Dipper. The Condor, 61(1), 4–17. 10.2307/1365341

[ece39696-bib-0018] Hartig, F. (2022). *DHARMa: Residual diagnostics for hierarchical (multi‐level/mixed) regression models* (R package version 0.4.6). https://cran.r‐project.org/package=DHARMa

[ece39696-bib-0019] Hendry, A. P. , & Berg, O. K. (1999). Secondary sexual characters, energy use, senescence, and the cost of reproduction in sockeye salmon. Canadian Journal of Zoology, 77(11), 1663–1675. 10.1139/z99-158

[ece39696-bib-0020] Hocking, M. D. , Dulvy, N. K. , Reynolds, J. D. , Ring, R. A. , & Reimchen, T. E. (2013). Salmon subsidize an escape from a size spectrum. Proceedings of the Royal Society B: Biological Sciences, 280(1753), 20122433. 10.1098/rspb.2012.2433 PMC3574346 23282994

[ece39696-bib-0021] Hocking, M. D. , & Reynolds, J. D. (2011). Impacts of salmon on riparian plant diversity. Science, 331(6024), 1609–1612. 10.1126/science.1201079 21442794

[ece39696-bib-0022] Hong, S. Y. , Wang, T. W. , Sun, Y. H. , Chiu, M. C. , Kuo, M. H. , & Chen, C. C. (2019). Stream type influences food abundance and reproductive performance of a stream specialist: The Brown Dipper (*Cinclus pallasii*). Journal of Ornithology, 160(1), 105–115. 10.1007/s10336-018-1604-6

[ece39696-bib-0023] Iida, M. , Yagi, Y. , & Iseki, T. (2021). Occurrence of wild chum salmon fry in the surf zone, and spawning and emergence timing in the adjacent nonstocked river in Niigata Prefecture, Japan. Fisheries Science, 87(4), 549–557. 10.1007/s12562-021-01535-4

[ece39696-bib-0024] IUCN . (2005). World heritage nomination—IUCN technical evaluation, Shiretoko (Japan) . https://whc.unesco.org/document/152000

[ece39696-bib-0025] Johnston, R. , Jones, K. , & Manley, D. (2018). Confounding and collinearity in regression analysis: A cautionary tale and an alternative procedure, illustrated by studies of British voting behaviour. Quality & Quantity, 52(4), 1957–1976. 10.1007/s11135-017-0584-6 29937587 PMC5993839

[ece39696-bib-0026] Kawaguchi, Y. , Taniguchi, Y. , & Nakano, S. (2003). Terrestrial invertebrate inputs determine the local abundance of stream fishes in a forested stream. Ecology, 84(3), 701–708. 10.1890/0012-9658(2003)084[0701:TIIDTL]2.0.CO;2

[ece39696-bib-0027] Kirillov, P. I. , Kirillova, E. A. , & Pavlov, D. S. (2018). Patterns of downstream migration of pink salmon *Oncorhynchus gorbuscha* in the Malaya Khusi River (Sakhalin Oblast). Journal of Ichthyology, 58(6), 889–901. 10.1134/S0032945218060085

[ece39696-bib-0028] Koshino, Y. , Kudo, H. , & Kaeriyama, M. (2013). Stable isotope evidence indicates the incorporation into Japanese catchments of marine‐derived nutrients transported by spawning Pacific Salmon. Freshwater Biology, 58(9), 1864–1877. 10.1111/fwb.12175

[ece39696-bib-0029] Kovach, R. P. , Gharrett, A. J. , & Tallmon, D. A. (2012). Genetic change for earlier migration timing in a pink salmon population. Proceedings of the Royal Society B: Biological Sciences, 279(1743), 3870–3878. 10.1098/rspb.2012.1158 PMC3415923 22787027

[ece39696-bib-0030] Kuzishchin, K. V. , Malyutina, A. M. , Gruzdeva, M. A. , Savvaitova, K. A. , & Pavlov, D. S. (2009). Reproduction ecology of masu salmon *Oncorhynchus masou* in the Kol basin (Western Kamchatka). Journal of Ichthyology, 49(6), 441–453. 10.1134/S0032945209060034

[ece39696-bib-0031] Levi, T. , Darimont, C. T. , MacDuffee, M. , Mangel, M. , Paquet, P. , & Wilmers, C. C. (2012). Using grizzly bears to assess harvest‐ecosystem tradeoffs in salmon fisheries. PLoS Biology, 10(4), e1001303. 10.1371/journal.pbio.1001303 22505845 PMC3323506

[ece39696-bib-0032] McMullen, L. E. , & Lytle, D. A. (2012). Quantifying invertebrate resistance to floods: A global‐scale meta‐analysis. Ecological Applications, 22(8), 2164–2175. 10.1890/11-1650.1 23387117

[ece39696-bib-0033] Minakawa, N. , & Gara, R. I. (2003). Effects of chum salmon redd excavation on benthic communities in a stream in the Pacific Northwest. Transactions of the American Fisheries Society, 132(3), 598–604. 10.1577/1548-8659(2003)132<0598:eocsre>2.0.co;2

[ece39696-bib-0034] Moore, J. W. , & Schindler, D. E. (2008). Biotic disturbance and benthic community dynamics in salmon‐bearing streams. Journal of Animal Ecology, 77(2), 275–284. 10.1111/j.1365-2656.2007.01336.x 18081781

[ece39696-bib-0035] Moore, J. W. , Schindler, D. E. , & Ruff, C. P. (2008). Habitat saturation drives thresholds in stream subsidies. Ecology, 89(2), 306–312. 10.1890/07-1269.1 18409419

[ece39696-bib-0036] Morita, K. (2021). Reverse migration of adult pink salmon (*Oncorhynchus gorbuscha*) to the sea after their return to fresh water. Environmental Biology of Fishes, 105, 1825–1832. 10.1007/s10641-021-01139-y

[ece39696-bib-0037] Munro, J. A. (1941). Studies of waterfowl in British Columbia: Greater scaup duck, lesser scaup duck. Canadian Journal of Research, 19(4), 113–138. 10.1139/cjr41d-010

[ece39696-bib-0038] Murata, S. (1900). First group of Hokkaido birds (2) (in Japanese). Zoological Magazine, 12(144), 365–369.

[ece39696-bib-0039] Nakamura, F. , & Komiyama, E. (2010). A challenge to dam improvement for the protection of both salmon and human livelihood in Shiretoko, Japan's third Natural Heritage Site. Landscape and Ecological Engineering, 6(1), 143–152. 10.1007/s11355-009-0083-6

[ece39696-bib-0040] Nakano, S. , & Murakami, M. (2001). Reciprocal subsidies: Dynamic interdependence between terrestrial and aquatic food webs. Proceedings of the National Academy of Sciences of the United States of America, 98(1), 166–170. 10.1073/pnas.98.1.166 11136253 PMC14562

[ece39696-bib-0041] Nilsson, A. L. K. , L'Abée‐Lund, J. H. , Vøllestad, L. A. , Jerstad, K. , Larsen, B. M. , Røstad, O. W. , Saltveit, S. J. , Skaugen, T. , Stenseth, N. C. , & Walseng, B. (2018). The potential influence of Atlantic salmon *Salmo salar* and brown trout *Salmo trutta* on density and breeding of the white‐throated dipper *Cinclus cinclus* . Ecology and Evolution, 8(8), 4065–4073. 10.1002/ece3.3958 29721280 PMC5916291

[ece39696-bib-0042] Obermeyer, K. E. , Hodgson, A. , & Willson, M. F. (1999). American Dipper, *Cinclus mexicanus*, foraging on Pacific salmon, *Oncorhynchus* sp., eggs. The Canadian Field‐Naturalist, 113(2), 288–290.

[ece39696-bib-0043] Obermeyer, K. E. , White, K. S. , & Willson, M. F. (2006). Influence of salmon on the nesting ecology of American dippers in southeastern Alaska. Northwest Science, 80(1), 26–33.

[ece39696-bib-0044] Ormerod, S. J. (1985). The diet of breeding Dippers *Cinclus cinclus* and their nestlings in the catchment of the River Wye, mid‐Wales: A preliminary study by faecal analysis. Ibis, 127(3), 316–331. 10.1111/j.1474-919X.1985.tb05073.x

[ece39696-bib-0045] Ormerod, S. J. , & Tyler, S. J. (1986). The diet of dippers *Cinclus cinclus* wintering in the catchment of the River Wye, Wales. Bird Study, 33(1), 36–45. 10.1080/00063658609476888

[ece39696-bib-0046] Ormerod, S. J. , & Tyler, S. J. (1991). Exploitation of prey by a river bird, the dipper *Cinclus cinclus* (L.), along acidic and circumneutral streams in upland Wales. Freshwater Biology, 25(1), 105–116. 10.1111/j.1365-2427.1991.tb00477.x

[ece39696-bib-0047] Ortlepp, J. , & Mürle, U. (2003). Effects of experimental flooding on brown trout (*Salmo trutta fario* L.): The River Spöl, Swiss National Park. Aquatic Sciences, 65(3), 232–238. 10.1007/s00027-003-0666-5

[ece39696-bib-0048] Pavlov, D. S. , Kirillova, E. A. , & Kirillov, P. I. (2008). Patterns and some mechanisms of downstream migration of juvenile salmonids (with reference to the Utkholok and Kalkaveyem rivers in Northwestern Kamchatka). Journal of Ichthyology, 48(11), 937–980. 10.1134/S0032945208110027

[ece39696-bib-0049] Pedersen, M. L. , Kristensen, E. A. , Kronvang, B. , & Thodsen, H. (2009). Ecological effects of re‐introduction of salmonid spawning gravel in lowland Danish streams. River Research and Applications, 25(5), 626–638. 10.1002/rra.1232

[ece39696-bib-0050] Polis, G. A. , Anderson, W. B. , & Holt, R. D. (1997). Toward an integration of landscape and food web ecology: The dynamics of spatially subsidized food webs. Annual Review of Ecology and Systematics, 28, 289–316. 10.1146/annurev.ecolsys.28.1.289

[ece39696-bib-0051] Quinn, T. P. (2018). The behavior and ecology of Pacific salmon and trout (2nd ed.). University of Washington Press.

[ece39696-bib-0052] R Core Team . (2022). R: A language and environment for statistical computing . https://www.r‐project.org/

[ece39696-bib-0053] Reimchen, T. E. (2017). Diverse ecological pathways of salmon nutrients through an intact marine‐terrestrial interface. The Canadian Field‐Naturalist, 131(4), 350–368. 10.22621/cfn.v131i4.1965

[ece39696-bib-0054] Romakkaniemi, A. , Perä, I. , Karlsson, L. , Jutila, E. , Carlsson, U. , & Pakarinen, T. (2003). Development of wild Atlantic salmon stocks in the rivers of the northern Baltic Sea in response to management measures. ICES Journal of Marine Science, 60(2), 329–342. 10.1016/S1054-3139(03)00020-1

[ece39696-bib-0055] Rundio, D. E. , & Lindley, S. T. (2008). Seasonal patterns of terrestrial and aquatic prey abundance and use by *Oncorhynchus mykiss* in a California coastal basin with a Mediterranean climate. Transactions of the American Fisheries Society, 137(2), 467–480. 10.1577/t07-076.1

[ece39696-bib-0056] Scheuerell, M. D. , Moore, J. W. , Schindler, D. E. , & Harvey, C. J. (2007). Varying effects of anadromous sockeye salmon on the trophic ecology of two species of resident salmonids in southwest Alaska. Freshwater Biology, 52(10), 1944–1956. 10.1111/j.1365-2427.2007.01823.x

[ece39696-bib-0057] Schindler, D. E. , Scheuerell, M. D. , Moore, J. W. , Gende, S. M. , Francis, T. B. , & Palen, W. J. (2003). Pacific salmon and the ecology of coastal ecosystems. Frontiers in Ecology and the Environment, 1(1), 31. 10.2307/3867962

[ece39696-bib-0058] Spiller, D. A. , Piovia‐Scott, J. , Wright, A. N. , Yang, L. H. , Takimoto, G. , Schoener, T. W. , & Iwata, T. (2010). Marine subsidies have multiple effects on coastal food webs. Ecology, 91(5), 1424–1434. 10.1890/09-0715.1 20503874

[ece39696-bib-0059] Takahashi, G. , Kuwahara, T. , & Yamanaka, M. (2005). Dams in the Shiretoko Peninsula: Issues in river management and environmental conservation (in Japanese with English abstract). Japanese Journal of Conservation Ecology, 10(2), 139–149. 10.18960/hozen.10.2_139

[ece39696-bib-0060] Taylor, A. J. , & O'Halloran, J. (1997). The diet of the Dipper *Cinclus cinclus* as represented by faecal and regurgitate pellets: A comparison. Bird Study, 44(3), 338–347. 10.1080/00063659709461069

[ece39696-bib-0061] Taylor, A. J. , & O'Halloran, J. (2001). Diet of dippers *Cinclus cinclus* during an early winter spate and the possible implications for dipper populations subjected to climate change. Bird Study, 48(2), 173–179. 10.1080/00063650109461215

[ece39696-bib-0062] Terui, A. , Negishi, J. N. , Watanabe, N. , & Nakamura, F. (2018). Stream resource gradients drive consumption rates of supplemental prey in the adjacent riparian zone. Ecosystems, 21(4), 772–781. 10.1007/s10021-017-0183-3

[ece39696-bib-0063] Thedinga, J. F. , Wertheimer, A. C. , Heintz, R. A. , Maselko, J. M. , & Rice, S. D. (2000). Effect of stock, coded‐wire tagging, and transplant on straying of pink salmon (*Oncorhynchus gorbuscha*) in southeastern Alaska. Canadian Journal of Fisheries and Aquatic Sciences, 57(10), 2076–2085. 10.1139/f00-163

[ece39696-bib-0064] Tonra, C. M. , Sager‐Fradkin, K. , & Marra, P. P. (2016). Barriers to salmon migration impact body condition, offspring size, and life history variation in an avian consumer. Ecography, 39(11), 1056–1065. 10.1111/ecog.02014

[ece39696-bib-0065] Wagner, M. A. , & Reynolds, J. D. (2019). Salmon increase forest bird abundance and diversity. PLoS One, 14(2), e0210031. 10.1371/journal.pone.0210031 30726212 PMC6364887

[ece39696-bib-0066] Walters, K. E. , Reynolds, J. D. , & Ydenberg, R. C. (2021). Ideal free eagles: Bald Eagle (*Haliaeetus leucocephalus*) distribution in relation to Pacific salmon (*Oncorhynchus* spp.) availability on four spawning rivers. Canadian Journal of Zoology, 99(9), 792–800. 10.1139/cjz-2020-0191

[ece39696-bib-0067] Whitehorne, I. (2010). Wintering behavior, physiology and site fidelity in a partial migrant, the American Dipper (*Cinclus mexicanus*). Waterbirds, 33(4), 461–470. 10.1675/063.033.0405

[ece39696-bib-0068] Wilcox, K. A. , Wagner, M. A. , & Reynolds, J. D. (2021). Salmon subsidies predict territory size and habitat selection of an avian insectivore. PLoS One, 16, e0254314. 10.1371/journal.pone.0254314 34237085 PMC8266124

[ece39696-bib-0069] Willson, M. F. , & Halupka, K. C. (1995). Anadromous fish as keystone species in vertebrate communities. Conservation Biology, 9(3), 489–497. 10.1046/j.1523-1739.1995.09030489.x

[ece39696-bib-0070] Wipfli, M. S. , Hudson, J. , & Caouette, J. (1998). Influence of salmon carcasses on stream productivity: Response of biofilm and benthic macroinvertebrates in southeastern Alaska, U.S.A. Canadian Journal of Fisheries and Aquatic Sciences, 55(6), 1503–1511. 10.1139/f98-031

[ece39696-bib-0071] Wipfli, M. S. , Hudson, J. P. , Chaloner, D. T. , & Caouette, J. P. (1999). Influence of salmon spawner densities on stream productivity in Southeast Alaska. Canadian Journal of Fisheries and Aquatic Sciences, 56(9), 1600–1611. 10.1139/f99-087

[ece39696-bib-0072] Yamada, T. , Urabe, H. , & Nakamura, F. (2022). Diel migration pattern of pink salmon fry in small streams. Journal of Fish Biology, 100(4), 1088–1092. 10.1111/jfb.15007 35129835

